# Acceptability of donated breast milk in a resource limited South African setting

**DOI:** 10.1186/1746-4358-6-3

**Published:** 2011-02-22

**Authors:** Irene Coutsoudis, Alissa Petrites, Anna Coutsoudis

**Affiliations:** 1 Department of Paediatrics and Child Health, University of KwaZulu-Natal, Durban, South Africa

## Abstract

**Background:**

The importance of breast milk for infants' growth, development and overall health is widely recognized. In situations where women are not able to provide their infants with sufficient amounts of their own breast milk, donor breast milk is the next preferred option. Although there is considerable research on the safety and scientific aspects of donor milk, and the motivations and experiences of donors, there is limited research addressing the attitudes and experiences of the women and families whose infants receive this milk. This study therefore examined attitudes towards donated breast milk among mothers, families and healthcare providers of potential recipient infants.

**Methods:**

The study was conducted at a public hospital and nearby clinic in Durban, South Africa. The qualitative data was derived from eight focus group discussions which included four groups with mothers; one with male partners; and one with grandmothers, investigating attitudes towards receiving donated breast milk for infants. There was also one group each with nurses and doctors about their attitudes towards donated breast milk and its use in the hospital. The focus groups were conducted in September and October 2009 and each group had between four and eleven participants, leading to a total of 48 participants.

**Results:**

Although breast milk was seen as important to child health there were concerns about undermining of breast milk because of concerns about HIV and marketing and promotion of formula milks. In addition there were concerns about the safety of donor breast milk and discomfort about using another mother's milk. Participants believed that education on the importance of breast milk and transparency on the processes involved in sourcing and preparing donor milk would improve the acceptability.

**Conclusions:**

This study has shown that there are obstacles to the acceptability of donor milk, mainly stemming from lack of awareness/familiarity with the processes around donor breast milk and that these could be readily addressed through education. Even the more psychological concerns would also likely be reduced over time as these educational efforts progress. With government and health care worker endorsement and commitment, breast milk donation could have a promising role in improving child health.

## Background

The importance of breast milk for infants' growth, development and overall health is widely recognized [1,2]. Moreover, breast milk is of special importance for preterm, low birthweight and other vulnerable infants [3].

The World Health Organization (WHO) recommends that for infants who cannot receive breast milk from their own mothers, the next preferred option is donated breast milk (donor milk) [4]. Expressed, pasteurized donor breast milk is not identical to fresh mother's milk, owing to some loss of micronutrients and anti-infective factors during pasteurization, decomposition over time, and normal variations in the makeup of breast milk. Still, enough of its bioactivity and immunological properties remain to ensure that - particularly when the gestational age of the donor's infant can be matched with that of the recipient infant - donated breast milk is superior to formula [5].

Although there is a substantial body of research on breast milk donation and banking, the bulk of this work has focused on the safety and scientific aspects of donor milk [6], milk banking policy [7], and the motivations and experiences of donors [8]. There has been only minimal recent research addressing the attitudes and experiences of the women and families whose infants receive this milk, with one paper focusing on breast milk donation in a Muslim context [9]. An earlier paper on this topic was written prior to the majority of research on the human immunodeficiency virus (HIV) and infant feeding, and also utilized different protocols for breast milk donation and banking [10]; as such, it is not applicable for the current situation.

As with any new health intervention, particularly one involving sensitive bodily fluids, determining its acceptability within the recipient community is a crucial first step. This is especially so in settings of high HIV prevalence, where various infant feeding choices are often stigmatized or feared because of their associations with HIV [11]. Ironically, it is in precisely these communities of high HIV prevalence that donated breast milk is most needed. The WHO and the United Nations Children's Fund (UNICEF) recommend that HIV-positive women should exclusively breastfeed for the first six months unless replacement feeding with formula is affordable, feasible, acceptable sustainable and safe (AFASS) [12]. As the majority of HIV-positive women in South Africa do not satisfy these criteria, exclusive breastfeeding is the recommended option to promote their infants' HIV-free survival. However, it is vital that this breastfeeding be exclusive, as mixed feeding carries a significantly higher risk of HIV transmission [13]. The availability of donated breast milk ensures that in the event that such women are temporarily unable to breastfeed, exclusive breastfeeding can be maintained.

Recognizing the importance of making donated breast milk available, one of the authors (AC) in conjunction with the head of the neonatal unit established a donor milk bank for low birthweight and other at-risk infants in the neonatal unit of King Edward Hospital, a resource-limited public hospital in Durban, KwaZulu-Natal, South Africa. However, anecdotal reports of fears and suspicions, historical beliefs regarding the desired characteristics of wet nurses [14], and the mistrust of healthcare services that is a legacy of apartheid policies [15] cast doubt on the acceptability of this practice. Given these concerns, as well as the aforementioned lack of research on acceptability, we sought to examine attitudes towards donated breast milk among mothers, families and healthcare providers of potential recipient infants. We also explored possible strategies for enhancing the acceptability of this practice.

## Methods

This research was carried out in the city of Durban, KwaZulu-Natal, South Africa. KwaZulu-Natal is one of the most resource-poor provinces of South Africa; in 2007 it had an official unemployment rate of 30%, the highest in the nation [16] and an infant mortality rate of 60 per 1000 live births [17]. It also bears the nation's heaviest burden of HIV/AIDS, with its 2007 antenatal HIV prevalence rate at 37.4% [16].

In seeking to obtain qualitative data on current attitudes towards donor milk and means of making breast milk donation more acceptable, we organised eight focus group discussions with various members of the community. Attitudes towards receiving donated breast milk for infants were conducted in four groups with mothers (referred to as M1, M2, M3 and M4), one with male partners (P), and one with grandmothers (G). We also conducted one group each with nurses (N) and doctors (D) about their attitudes towards donated breast milk and its use in the hospital. This breakdown was intended to be representative of the various groups involved in infant feeding decisions and practices, with the most attention paid to the mothers given their primary role. Family involvement was assumed to be an important factor, hence the inclusion of the grandmothers and partners.

Participants were purposefully selected either through patient records of a local clinic or because they were boarding or working in King Edward Hospital, a typical South African public hospital that serves a low-income, primarily Zulu population. The clinic was located in Cato Manor, a township with both informal and formal housing, which is the closest source community to the hospital. The doctors and nurses were recruited through word of mouth by the investigators, and all other participants were recruited through direct approach or telephone call by two Zulu-speaking research assistants. The focus groups were carried out in September and October 2009.

Each group had between four and eleven participants, leading to a total of 48 participants for the study as a whole. Demographic information for each group is displayed in Table 1.

**Table 1:** Characteristics of participants

Group	Number	Mean age in years	Mean No. of children	Mean educational level reached	No. Employed	No. Single
Mothers (M)	20	28	2	Grade 10	3	18
Grandmothers (G)	5	56	7	Grade 3	0	1
Partners (P)	4	34	3	Grade 9	3	4
Nurses (N)	8	42	2	Tertiary	8	6
Doctors (D)	11	28	0	Tertiary	11	4

Each focus group discussion began with collecting demographic information on the participants, explaining the purpose of the study, and obtaining informed consent. All discussions were conducted in Zulu (by a trained interviewer) except the groups with the nurses and doctors, which were conducted in English. The focus groups were carried out with a largely structured format; for each group, we developed a set of questions that the moderator used to guide the discussion, probing for more information or clarification when necessary. The groups were documented by tape-recording as well as hand note-taking by at least one observer who understood the language of discussion. Following each group, tape-recordings were translated into English (when necessary) and simultaneously transcribed.

Transcripts were analyzed using qualitative content analysis and coding techniques as described by Graneheim and Lundman [18] and Ulin and colleagues [19]. All sections of the transcripts that were relevant to the study objectives were categorized based on subject matter and were then collected under these headings; each of these categories were analyzed for themes and recurring concepts, which were then used to structure the final write-up.

This study was approved by the Biomedical Ethics Committee of the Nelson R. Mandela School of Medicine, University of Kwa-Zulu Natal (BE176/08). Written informed consent was obtained from each participant.

## Results

The participants discussed ideas about infant feeding and mentioned the importance of breastfeeding, as well as the factors that result in lower than expected levels of breastfeeding. Issues around wet nursing and formula feeding highlighted the influences these could have on breastfeeding. The majority of the discussion centered around obstacles with regards to the acceptability of donor breastmilk. The issues that emerged around the acceptability of donor breastmilk were: concerns for its safety; lack of familiarity with its use; and discomfort and sensitivity of using a bodily fluid from another person.

### Ideas about infant feeding

#### I. Significance of breast milk

Given that general ideas about infant feeding are crucial to decisions of whether or not to accept donor milk, participants were first asked about these opinions. Most of the participants felt that breast milk is better than formula, and that the ideal way of feeding a baby is breastfeeding. The reported benefits of breastfeeding included its nutritional properties, protection against disease, affordability, convenience, warmth, and its role in bonding.

From these responses, it is clear that most participants appreciate the importance of breast milk and its role in promoting infants' health and well-being.

#### II. Prevalence of breastfeeding

Another noteworthy point in the discussions on general infant feeding issues was the low prevalence of breastfeeding. Of those asked how common it is to breastfeed, the majority stated that it is no longer a common practice. The "diseases" of the current era were given as the primary reason for this drop in breastfeeding rates: as stated by one mother,

"because of the disease that are around these days, breastfeeding is not common anymore." (M2:5)

Although few of the participants actually identified HIV by name, an issue that will be further discussed in the stigma section, it was clear that this was the primary disease to which they were referring. While it is not surprising that breastfeeding rates would have been altered by the advent of HIV, what emerged in these discussions, and also later in conversations on wet nursing, is that the breastfeeding landscape has been fundamentally changed by HIV. Other reasons given for the decrease in breastfeeding rates included societal changes such as the beliefs that breastfeeding is impractical for working women, no longer fashionable, or associated with the lower classes.

#### III. *Wet nursing*

Given that wet nursing may serve as cultural precedent to breast milk donation, participants were also asked about their beliefs on this topic. Most reported that they had heard of wet nursing. It was noteworthy that several participants made the connection between wet nursing and breast milk donation themselves. One of the nurses commented that

"even in our history, we're talking about our culture [motions to the woman next to her] if she's still having the scanty supply, I'm having plenty, [motions to someone else], she's having plenty, her baby cries and she's going to toilet so I'll just take her baby and give, and breastfeed the baby. I think to replace that culture, though there are still many complications, I think using donor milk is ideal." (N2)

However, as with the decrease in overall breastfeeding rates, participants reported that wet nursing has also been deeply changed by HIV, and commented that wet nursing is either no longer practiced or no longer "right" in current times, almost invariably citing the threat of disease transmission. One mother stated that:

*"in these days no one is wet nursing because of the diseases we have," (M1:5) *and another noted that:

"a long time ago, it was right. But not now, due to these diseases. Before, there were diseases like TB but it can be cured, but the diseases today have no cure. So wet nursing today is totally wrong. It mustn't happen now." (M4:4)

Again, though most participants used the general term "diseases," this generally seemed to be in reference to HIV.

A final issue raised in relation to wet nursing was the fear of the infant bonding with the wet nurse. One of the mothers remarked that:

"if I breastfeed somebody's baby that baby's going to make a bond with me, otherwise if my baby is getting somebody else's milk it's going to bond with that somebody." (M1:5)

#### IV. Infant formula

The discussions suggested a wide variety of ways in which formula and formula marketing has affected breastfeeding and now breast milk donation.

Firstly it became clear that beliefs about the advantages and disadvantages of formula are intimately related to corresponding ideas about breast milk. A few participants expressed concerns about the safety and risks of infant formula. One of the nurses noted that caregivers often mix formula incorrectly (N3). Similarly, one of the partners commented that formula is not guaranteed to be 100 percent pure, and that infant follow-on cereals come from overseas and may even be expired by the time they arrive in South Africa (P1). However, as seen in the concerns about the safety of breastfeeding in the context of HIV, most participants implicitly believed formula to be the safer alternative. While true that formula does not carry the threat of HIV-transmission, in resource-poor settings the other hazards of formula, particularly the risks of diarrhea and malnutrition, generally outweigh the threat of HIV [12]. Formula marketing also played a major role in the acceptability of donor milk. For instance, several of the participants reported that donated breast milk would be more acceptable if it was brought to them in appealing containers. One of the mothers went so far as to make explicit the connection between this desire and the packaging of formula:

"If we can teach pregnant women about this and try to put it in nice, attractive containers, then people will accept it and disregard formula." (M1:1)

### Obstacles to accepting donated breast milk

#### I. Fears about donated breast milk

The most commonly and explicitly stated drawback to the acceptability of donated breast milk was fear about its safety, most notably its risk of containing HIV. Participants frequently commented that they were afraid that their baby might become infected with HIV through donor milk. According to one mother,

"they'll be worried that the baby might get diseases from that milk."(M4:1)

Similarly, those who said that they would accept donated breast milk often made this contingent on certain safety standards being met. One partner stated,

"I don't have any problem, as long as they're going to check the condition of that somebody who's donating the milk, and that it's clean and 100% checked." (P3)

The first explanation for this fear is a basic lack of awareness about the process of breast milk donation. Participants were either explicitly or implicitly unaware of the fact that donors are screened for HIV and other infectious diseases, and that their milk is then pasteurized, which destroys HIV and other pathogens and thus acts as an additional check. For instance, one of the mothers posited that

"the reason that other women aren't going to accept it is because they don't know where it's tested, how it's tested, is it tested?" (M4:4)

Beyond this lack of awareness, though, was a deep-seated lack of trust about the efficacy of these processes. Many participants did not trust the screening procedures to identify appropriate donors, as with one nurse who commented that

"I don't believe the screening is 100%." (N6)

This was often clarified as a concern about the window period of the HIV test: the period of time from HIV infection until a test can detect any change. Though several safeguards are in place to ensure that donors are not in a window period - requiring more than one HIV test, and screening for lifestyle factors that would suggest a higher likelihood of infection - participants still expressed doubt. As stated by another nurse,

"I'm not happy [about the window period]." (N2)

An associated issue was participants' lack of trust in the pasteurization process. Throughout the discussions, participants expressed a desire to see or learn about the steps of the pasteurization process. One mother declared that she would accept donated milk ...

"... as long as they explain to me about donated breast milk. The whole process that it's gone through, who donated milk, if it was pasteurized, if it's safe. I would want to know all those things." (M2:4)

Similarly, one of the partners explained that

"we must show [the mothers] all the procedures, and do it practically. Not to sit on the table or in the class and just show them on a paper. We must show them where it's coming from, where it stays, the whole procedure." (P1)

In addition to those concerns specifically related to donor breast milk, expressions of mistrust occurred in a number of contexts, all of which are likely intertwined and may combine to produce a general sense of skepticism. A lack of trust was voiced in relation to the HIV test as well as healthcare services and personnel as a whole. As discussed above, the possibility of receiving a false negative result during the window period is a reality of the ELISA test, and this has direct repercussions for the perceived safety of donor milk. However, it may also be affecting attitudes toward breast milk donation - and healthcare services in general - in a more subtle way. What is simply an unfortunate feature of an otherwise functional test may in some cases be interpreted to mean that the test is faulty or ineffective, which may then breed a broader feeling of mistrust. For example, one grandmother commented that if her daughter were sick and could not breastfeed her own baby,

"I would accept it. But I wouldn't like it, because of you doctors. You test people, you tell them that they don't have diseases. [Describes her experience of being tested for high blood pressure.] But now I'm old they're telling me that I do have this." (G3)

While these participants' experiences and beliefs were not necessarily founded in medical errors, they have been interpreted as such, and this has likely had ramifications for their faith in the healthcare system as a whole.

Although not referencing any past malpractice or mistakes, other participants also communicated a lack of trust in healthcare providers. One partner, when asked whether it matters who prescribes or delivers the milk, stated that

"no, there's no difference. It can be a doctor or a counsellor, because you cannot be sure how careless they are; both of them. You cannot trust both of them." (P1)

Despite this widespread lack of trust, other participants did express slightly more faith in healthcare services. One mother asserted that

"there are no fears about donated breast milk, because I'll be in the hospital, given the milk by someone who knows, and it's tested. I would trust the people who are working there in the hospital." (M4:4)

Another mother also suggested that

"I don't think there will be any fears about breast milk as long as it's lab-tested." (M1:1)

Among those who did communicate faith in healthcare services, this was often tied to the concepts of hospitals or laboratories, suggesting that these institutional images may carry more perceived legitimacy than individual healthcare providers.

Beyond this lack of trust and awareness, discussions about the safety of donor milk remain hampered by the still-fiery stigma surrounding HIV. This was evident in a number of contexts: the mistrust and issues with disclosure discussed above, and, perhaps most prominently, people's refusal to identify the disease by name. On several occasions participants referred to "diseases" of the current era, and when probed for specifics would give examples such as breast cancer or "BP" (high blood pressure). Whether they actually believed that conditions such as breast cancer and high blood pressure can be transmitted through breastfeeding is uncertain, but the noteworthy point was the common refusal to say the word 'HIV'. In one particularly striking example, one of the nurses - speaking about wet nursing - stated that

"there are many diseases. It's no more practiced. I know it has been practiced before, but now it's no more practiced because of [motions quotation signs with her hands.]" (N2)

This stigma is something that must be addressed on a broad, community-wide level, for it has far-reaching implications well beyond the practice of breast milk donation. As it was poignantly put by one mother,

"we need to educate the moms because some moms aren't going to accept it because they're afraid of diseases. So we need to inform them, and also teach them that if you're HIV-positive you're not like an animal. You're still a human being." (M3:5)

A final fear regarding donated breast milk - again tied in with a lack of awareness - was a basic fear of the unknown. One mother believed that people would be unwilling to accept donor milk

"because we are not well trained about this donor milk. No one has talked about it to us and so most of the time people are scared to take it." (M3:5)

Fortunately, this issue is one of the most easily addressed, and basic education about the practice of breast milk donation will likely alleviate many of these fears.

In addition to wanting to observe the pasteurization, several participants mentioned that they were either uncomfortable not knowing - or wanted to know - the identity of their baby's breast milk donor. One doctor remarked that

"if I knew who was donating that breast milk, like if it was someone who was known to me, I'd feel more comfortable choosing that over formula, even if it was just a colleague or something. Because there's still that uncertainty: has this cleaning process been absolutely effective?" (D2)

A related issue - which was only raised by one participant in this study, but which has been discussed elsewhere in the literature on breast milk donation - is the possibility that the donor's identity is relevant even beyond logistical issues such as her health. The requirement that the recipient know the donor has been noted in one paper about breast milk donation in Kuwait, but in this situation this stipulation was based on Muslim ethics [9]. The authors of this paper facilitated the process of breast milk donation by arranging for the donor to meet the recipients, but the data presented are for only three cases studies; given the much larger scale of breast milk donation in our setting, arranging this type of contact would be infeasible. The only instance of this sentiment - wanting to know the donor for reasons other than safety - came from one of the mothers, who stated that

"my other fear is that women talk amongst themselves, and if you tell someone that your baby's getting donated breast milk and later when the baby's older they tell him, he'll want to know what that mother was." (M1:1)

As this concern was raised by only one participant in one of the focus groups, it is difficult to assess its significance in this community.

#### II. Lack of familiarity

Very few participants had heard of breast milk donation. However, awareness was greater among the mothers in King Edward Hospital (where breast milk donation was actually being practiced) compared to those from the Cato Manor Clinic. With regards to experience, two participants in two separate mothers groups had been given donor milk. Given that only a few of the participants had received any exposure to the idea or practice of breast milk donation, the initial resistance expressed by some is not surprising. It is more than reasonable to expect that some prior knowledge of, or experience with an intervention would be a necessary prerequisite for accepting it. As put by one of the doctors, reflecting on her own hesitancy to accept donor milk,

"I think maybe just getting around this whole idea of donor milk is a fairly new thing." (D4)

The importance of having familiarity and ideally experience with breast milk donation was clearly illustrated by the fact that those participants who had been exposed to the practice were generally more convinced of its value and efficacy. One mother who had received donated breast milk for her baby declared that

"it's made a difference in my baby. While I was sick in high care they gave donor milk and my baby gained weight quickly." (M2:2)

Even secondhand exposure can be beneficial, as later in this same focus group another mother commented,

"I think it's working because it worked for Mom #2 when she was in high care." (M2:5)

Perhaps the most evidence-rich comment came from one of the nurses, who affirmed that

"we're using donor milk. It's helping them. It is a good thing with the neonates, especially the prem[ature] babies. It has got good outcomes." (N8)

One of the doctors also told a story of nurses who - having seen the effects of donor milk - began actively seeking it out and asking that it be given to certain vulnerable babies. (D1)

Another demonstration of the impact of familiarity was that several participants mentioned wanting to meet or see examples of mother-baby pairs who had already used donated breast milk. One mother suggested that

"if we can maybe involve a mom who already received the donor milk and whose baby grew well if we have her as an example that might help." (M3:5)

#### III. Discomfort and sensitivity

Beyond a sense of unease with expressed breast milk was an aversion to the fact that it was a bodily fluid from another person as communicated by one of the doctors,

"it's just that it's milk from someone else and it's not artificially made, it's from someone else. It's just an uneasy feeling, and it's the way I feel." (D4)

A feeling of sensitivity also surfaced in one discussion regarding race. Participants were asked whether they felt the race of the breast milk donor to be important. In seven of eight groups, they firmly responded that the race or ethnicity of the donor was insignificant. As put by one mother,

"we're all the same. Even our breast milk is the same. The race doesn't matter because we're all the same." (M3:5)

In some cases participants related the question to blood donation, saying that race does not matter in either case. One grandmother observed that

"even the blood is the same. It doesn't depend on skin color." (G1)

Another mother turned the question back to the issue of safety, saying that

"what matters is if you're healthy or not healthy. It doesn't matter who donated the breast milk, as long as you're healthy." (M2:4)

Given the strong negative response from the vast majority of the groups, it came as a surprise when the same question was posed to the nurses' group and four out of eight participants immediately responded that they would not accept breast milk donated by a woman of a different race. When probed further, they gave a variety of explanations. One nurse clarified that it was not an issue with clan (Zulu vs. Xhosa, for example) so much as the color of the skin (N8). Two others said that they would prefer breast milk donated from people in the local community as opposed to foreigners, but without any specific reference to race (N4, N5). In a different vein, a conversation among several nurses suggested that people of different races live different lifestyles and have different diseases, and that white donors are more likely to get tattoos, be smokers, and have "Caucasian diseases". Based on this line of reasoning, it appears that their issue with skin colour is not so much a psychological issue but rather a concern with safety. It is therefore important that potential recipients should be reminded that donors are screened for precisely the kind of lifestyle characteristics mentioned above. However, in cases where there are psychological issues with skin color, the nurses suggested that rather than giving information about the donors, the conversation should be redirected towards emphasizing the scientific benefits of donor milk.

"it's nice in this way we way explain what is donor milk, what are the advantages of donor milk to this baby, why are we encouraging her to use this donor milk?" (N8)

Another noteworthy point regarding breast milk donation and race was that two nurses said that they would accept blood from a white donor but not breast milk. The first explained that

"blood is the same," (N4)

whereas the second reasoned that

"blood is just for a few hours." (N5)

This second statement closely echoed a comment by a mother who remarked,

"I'll just tell myself that if the donor milk were for a short time, then I can accept it." (M1:2)

This issue of blood generally being a short-term intervention whereas donor milk is more sustained, and participants' complaints that they would have to sit and look at it, suggests a certain degree of sensitivity with breast milk that is perhaps absent with blood.

## Discussion

Safety issues were voiced as important concerns by the majority of the participants as were issues of lack of familiarity with the concept of breastmilk donation and the procedures involved. There was also suggestion of discomfort around the issue of giving breastmilk from another mother and not from the mother herself. Although it was not stated by any of the mothers themselves, it is certainly probable that women could feel uncomfortable and even ashamed about their inability (even if only temporarily) to feed their own babies, and that this could produce a greater sensitivity about donor milk. Given that this is a possibility, education should emphasize that an inability to produce milk is not a personal shortcoming but rather a medical circumstance, and that these women can still provide their babies with the love and care that are the essence of mothering.

The need for education regarding donor milk emerged as a dominant consideration and was explicitly raised by participants in each of the focus groups. When asked how donated breast milk could be made more acceptable, education was nearly always the first strategy mentioned. As stated by one of the mothers,

"before we start anything about donor milk we need to educate people, because people won't accept things easily. Everyone must be made to understand." (M1:2)

The fact that this is being asked for by the community - as opposed to imposed from the top down - conveys that there is a willingness and desire to learn and that the community will likely be receptive to these efforts.

The question of where this education should occur sparked some debate among participants. Education in the hospital for actual recipient mothers is crucial; in this study, this was illustrated quite well by the frustration and confusion of one mother who had not received any education prior to her baby being given donor milk:

"when I came the first time to see the baby, the baby was already receiving this milk." [So when they came did they explain it to you?] "No, they didn't explain it. They just showed me the room where I was supposed to get the milk. But they didn't explain anything about what it is, where they're getting it from." (M3:3)

While hospital-based education is undeniably important, participants also emphasized that education must occur antenatally. One of the nurses commented that,

"they [must start with] ANC training. They should be teaching the mothers antenatally about breast milk, doing all what you are doing here. Encouraging them to understand what is donor milk, so that it hasn't got that [label] as a foreign something. People are familiar with the word even if they didn't see, when they come in, 'this is the donor milk you were taught about.'" (N8)

Learning about breast milk donation early on and without the emotional stress of having to make an immediate decision would help to ease a number of the constraints discussed previously. One of the reasons that the nurses group in particular supported the idea of antenatal education was their belief that with their busy schedules, it would be impossible to conduct widespread education about breast milk donation in the hospital itself.

Beyond discussing education for mothers alone, participants suggested that this education should be extended to family members and the broader community as well. Family members' influence on the acceptability of donor milk was assumed to be an important factor and therefore was built into the design of this study, hence the groups with the grandmothers and partners. The discussions confirmed that family members do indeed influence women's decisions and beliefs about infant feeding, but that the problems that occur often arise out of a simple lack of education.

Among the mothers and the also grandmothers themselves, several participants communicated the importance of educating grandmothers. One mother explained that

"the grannies need to be told what is happening these days. In the past there were no diseases and so [behaviors were different], and now they're getting information from all sorts of different people. And now the grannies are scared about these diseases. Even if I'm not at home and I express my breast milk, she'll be more understanding because she'll have heard this information." (M2:5)

Although this idea was less common in relation to the partners, one did suggest that

"if you can teach [the mothers], they're the ones who are going to tell us at home. But we need to be taught, but we don't have time." (P3)

Except for this partner, none of the participants offered specific ideas on ways of reaching family members and carrying out this education. However, one noteworthy comment on the scope of education came from one of the doctors. When asked whether she felt that providing donated breast milk is sustainable, she replied,

"yes, if it's advocated not just in our setting but through government and media. It's actually fashionable." (D2)

This suggestion for a large-scale campaign to promote breast milk donation and donor milk (though possibly provoking skepticism at first) may in fact be a highly productive maneuver. While it is true that donated breast milk is utilized by only a small proportion of the population, educational efforts regarding donor milk are best incorporated into broader campaigns to protect, promote and support breastfeeding; given breastfeeding's vital role in promoting infant and child health [2], such campaigns can have far-reaching public health impacts. As this doctor pointed out, the crucial factor in whether such campaigns succeed is government's involvement and stamp of approval. This is best exemplified in the case of Brazil, where the government's support and oversight of breast milk donation and breastfeeding has resulted in enormous growth in breast milk donation and banking in the past 20 years [20]. This has produced significant improvements in public health outcomes: between 1975 and 2003, Brazil's under-five mortality rate dropped from 136/1000 to 20/1000 [14]. The parallels between Brazil and South Africa are more than enough to suggest that a similar effort could be highly successful in this setting as well. With crucial government endorsement and support, this intervention has enormous potential for improving the health and wellbeing of South Africa's infants and children and ultimately society as a whole.

Related to the issue of education by health professionals an issue that emerged which needs consideration is of mistrust of health professionals and the health system. These various expressions of mistrust need to be addressed as they have implications far beyond those related to acceptability of breast milk donation. In all breastmilk bank operations efforts must be made to emphasize the various precautionary efforts taken with donor milk in order to develop a level of trust and transparency with the mothers and families. The request by some participants for the identity of the donor to be revealed as a further element of transparency and trust is generally not an option. However, creating educational materials that illustrate the stepwise process of screening, donation, pasteurization and storage should address this concern. Providing potential recipients with as much information as possible is likely to be the best strategy for strengthening their confidence in the safety of breast milk donation.

Cultivating trust in healthcare professionals - and in the efficacy of the healthcare system as a whole -is a far more nebulous and long-term challenge. It is widely recognized that the poor quality of care offered in public healthcare services has had far-reaching implications for the nation's health, as well as for social stability and economic growth [21]. Though daunting, instituting enduring efforts to raise the standard of care in public clinics and hospitals is vital; this would engender trust and satisfaction in a wide range of healthcare programs, including, but certainly not limited to donor milk banking, and would ultimately result in greater health for all.

## Conclusion

This study has shown that there are obstacles to the acceptability of donor milk, mainly stemming from lack of awareness or familiarity and that these could be readily addressed through education. Even the more psychological concerns would also likely be reduced over time as these educational efforts progress. While this research has only utilized a small sample size from one community, it is our belief that these results can be generalized to most of the populations served by South African and other public hospitals. Thus, with government and health care worker endorsement and commitment, breast milk donation could have a promising role in improving child health.

## Competing interests

The authors declare that they have no competing interests.

## Authors' contributions

IC conceived the study and all authors contributed towards planning and design of the study. IC and AP were observers in the focus groups that were conducted in Zulu and they facilitated the focus groups that were conducted in English. IC and AP were responsible for data collation and analysis. All authors contributed to writing and read and approved the final manuscript.
